# Three-Dimensional Tracking of Small Aquatic Organisms Using Fluorescent Nanoparticles

**DOI:** 10.1371/journal.pone.0078498

**Published:** 2013-11-07

**Authors:** Mikael T. Ekvall, Giuseppe Bianco, Sara Linse, Heiner Linke, Johan Bäckman, Lars-Anders Hansson

**Affiliations:** 1 Aquatic Ecology, Department of Biology, Lund University, Lund, Sweden; 2 Biochemistry and Structural Biology, Department of Chemistry, Lund University, Lund, Sweden; 3 Nanometer Structure Consortium (nmC@LU) and Solid State Physics, Lund University, Lund, Sweden; 4 Centre for Animal Movement Research, Department of Biology, Lund University, Lund, Sweden; RMIT University, Australia

## Abstract

Tracking techniques are vital for the understanding of the biology and ecology of organisms. While such techniques have provided important information on the movement and migration of large animals, such as mammals and birds, scientific advances in understanding the individual behaviour and interactions of small (mm-scale) organisms have been hampered by constraints, such as the sizes of existing tracking devices, in existing tracking methods. By combining biology, chemistry and physics we here present a method that allows three-dimensional (3D) tracking of individual mm-sized aquatic organisms. The method is based on *in-vivo* labelling of the organisms with fluorescent nanoparticles, so-called quantum dots, and tracking of the organisms in 3D via the quantum-dot fluorescence using a synchronized multiple camera system. It allows for the efficient and simultaneous study of the behaviour of one as well as multiple individuals in large volumes of observation, thus enabling the study of behavioural interactions at the community scale. The method is non-perturbing – we demonstrate that the labelling is not affecting the behavioural response of the organisms – and is applicable over a wide range of taxa, including cladocerans as well as insects, suggesting that our methodological concept opens up for new research fields on individual behaviour of small animals. Hence, this offers opportunities to focus on important biological, ecological and behavioural questions never before possible to address.

## Introduction

Tracking the motion and migration of individual organisms is of crucial importance for understanding their biology and ecology. Tracking of larger organisms such as mammals, birds and fish is relatively straightforward using well-established techniques and equipment such as radio- and global positioning (GPS) collars [1], [2] and passive radio frequency identification (RFID) transponders (PIT tags) [3], [4]. However, tracking of smaller, and in particular aquatic organisms, poses three considerable challenges.

First, the size and weight of currently available transponders are a limiting factor to studying mm-scale organisms; the smallest tracking transponders available today are about 0.4 mm in size [5], which means that these devices drastically affect the behaviour of organisms at the mm length-scale. The size-limitation of current tracking devices has therefore precluded studies focusing on movement of smaller organisms, such as crustacean zooplankton, in aquatic environments.

Second, in contrast to land-based, flight-less organisms, in which case a two-dimensional (2D) tracking approach is often satisfactory, tracking of aquatic organisms requires following organisms’ movement in a three-dimensional (3D) environment; to obtain accurate information regarding the position and speed of individual organisms, and allow reliable distinction between behaviours such as cruising and sinking [6].

Third, to obtain information on interactions between organisms requires studying the organisms in larger environments. So far, tracking of multiple mm-scale organisms in 3D has, to our knowledge, been limited to volumes of observations of 1 litre [7], [8]. Using larger volumes of observation allows a researcher to simultaneously overlook a larger volume and, hence, more organisms, allowing for research also on the interactions between groups of organisms.

Here we present a tracking method that overcomes all of these challenges and enables the non-perturbative tracking of the individual motion and behaviour of multiple small, mm-sized, organisms, in 3D, in large volumes of observation. The method is based on 1) a new refined method for labelling organisms with fluorescent nanoparticles, so-called quantum dots, and 2) a 3D tracking approach based on capturing the fluorescent signals from the excited quantum dot using a synchronized multiple camera system. The labelling does not affect the behavioural response of the studied organisms and is generally applicable to a wide range of organisms; we provide proof-of-principle demonstrations of tracking for *Daphna magna*, ostracods and two types of aquatic insect larva, a mayfly larvae (*Cloeon sp.*) and a *Chaoborus* larva. This method has the potential to significantly advance our understanding of the biology and ecology of small organisms and to answer questions regarding the behaviour of small organisms that were previously impossible to address due to methodological constraints, for example individual behavioural decisions and interactions within and between populations or communities. In a broader perspective we suggest that this new technique will advance research on plankton ecology and that the use of quantum dots for tracking is also applicable for tracking small organisms not only in water, but also in air and on land.

## Methods

Our tracking approach relies on a new refined method for labelling organisms using quantum dots. Quantum dots, which are commercially available (e.g. http://www.lifetechnologies.com/), have been used for a wide range of applications because of their fluorescent properties and high photostability [9]; areas of use have included e.g. *in-vivo* and *vitro* biomedical imaging [10]–[13] and 2D tracking of organisms [14]. To ensure that the quantum dots adhere to the organism we first coat them with poly-L-lysine, a small natural homopolymer of the amino acid L-lysine, see below.

### Coating of Quantum Dots

The quantum dots were conjugated with poly-L-lysine in order for them to associate with the zooplankton. 50 µL of 585 ITK Carboxyl quantum dot at 8 µM (yellow) or 655 ITK Carboxyl quantum dot at 8 µM (red) (Life technologies, Prod. Nr.: Q21311MP and Q21321MP respectively) were added to 400 µL of 10 mM borate buffer, pH 7.4 and mixed briefly on a vortex machine. After this, 4 mg of poly-L-lysine hydrobromide (Sigma Aldrich, Prod. Nr.: P6516) dissolved in 80 µL of 10 mM borate buffer, pH 7.4, was added to each tube and the solution was mixed briefly on a vortex machine. After this, 46 µL of 10 mg/mL of N-(3-Dimethylaminopropyl)-N'-ethylcarbodiimide hydrochloride (EDC) was added to each tube and the solution was mixed briefly on a vortex machine. The tubes were incubated in darkness at room temperature (approx. 21°C) for 80 minutes and the solution was mixed several times during the incubation by inversion of the tubes. In order to remove excess poly-L-lysine, the quantum dot solution was transferred to a PES centrifugal ultrafiltration unit with a 100 kDa cut-off (Vivaspin 500) and centrifuged for two minutes at 13,000 RPM. The retentate was washed six times with 100 µl of 50 mM borate buffer, pH 8.3 in the centrifugal device. After the last centrifugation, the poly-L-lysine-conjugated quantum dots were resuspended in 50 mM borate buffer, pH 8.3 to a final volume of 576 µl and stored in darkness at 4°C until zooplankton labelling.

### Zooplankton Labelling

The zooplankter species *Daphnia magna* is a common freshwater species with an adult size range of 1–2 mm. Prior to labelling, three *D. magna* were transferred together with water from a culture aquarium to a 2 mL tube. Most of the water from the culture aquarium was removed by aspiration and 250 µL of water was added (N.B. always leaving a thin film of water covering the animals). The poly-L-lysine conjugated quantum dot solution (40 µL, prepared as above) was added and the content mixed carefully using a pipette. The solution was incubated at room temperature (approx. 21°C) in darkness for one hour. During the incubation the poly-L-lysine-coated quantum dots adhere to the surface of *D. magna* because of the non-covalent interaction between the positively charged polyelectrolyte poly-L-lysine and the negatively charged carapace of *D. magna* - this mechanism is similar to the one used in layer-by-layer deposition methods for coating of charged particles [15]. After the incubation most of the quantum dot solution was removed by aspiration and the *Daphnia* were rinsed three times by carefully adding and removing 1 mL of water using a pipette, always leaving enough solution in the tube to cover the *Daphnia* in order for them to not be exposed to air, which could be fatal. After labelling, the *Daphnia* were kept according to labelling colour in 50 mL of water in 100 mL jars awaiting trials.

### Fluorescence Microscopy

In order to monitor where the quantum dots associated with the *Daphnia* we placed one individual under an inverted fluorescence microscope (Nikon Eclipse TS100) at 100 times magnification. Any quantum dots present on the *Daphnia* could be detected when excited by the fluorescent light (465–495 nm). A microscope camera (Nikon DS-Fi1) mounted on the microscope was used to obtain pictures of the labelled *Daphnia*.

### Cameras

**Figure 1:**
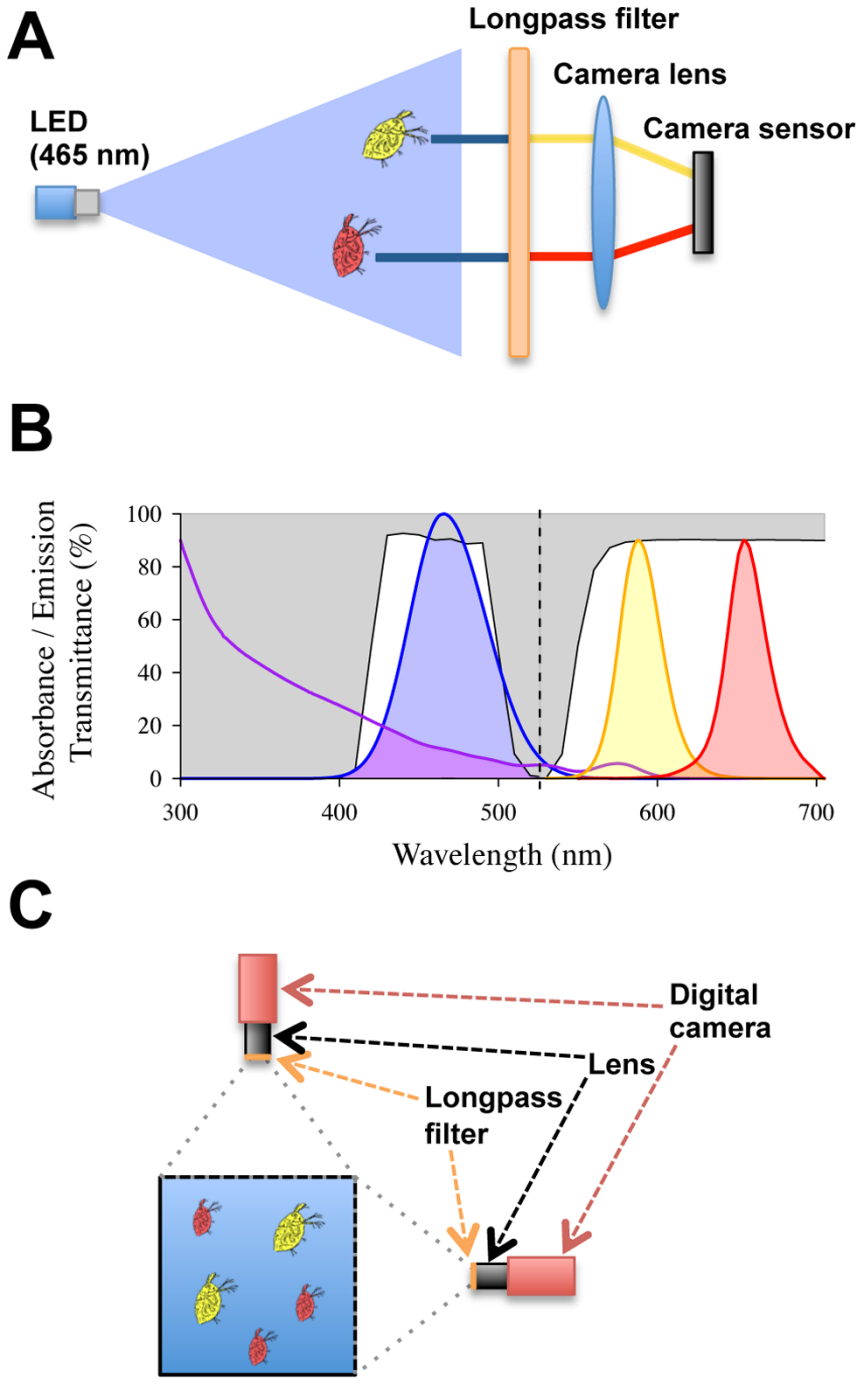
Schematic illustration of fluorescence system. (a) Schematics of the tracking setup. Light emitted from the excitation source (a light-emitting diode array – LED array) causes the quantum dots to fluoresce. The emitted fluorescence is filtered and focused on a camera sensor. (b) Quantum dots are excited in the emission range of a blue LED array (blue line) filtered by a bandpass filter to out light of unwanted wavelengths (white area, left side of the panel). The blue area represents the light available for the excitation; the light absorbed by the quantum dots is represented by the purple area below the quantum dot absorbance spectrum (purple line). The emission spectrum of the quantum dot fluorescent at 585 nm and 655 nm (yellow and red lines, respectively) are filtered with a longpass filter (white area, right side of the panel) before being recorded by the camera sensor. All the curves are normalized versus the excitation light (peak at 465 nm). (c) Illustration of camera and aquarium set-up seen from above, showing the placement of, cameras, lenses, camera filters and aquarium.

To record the positions of the animals we used two Pike F-210C colour cameras (Allied Vision Technologies GmbH, Stadtroda, Germany) equipped with a 1 inch, 1920×1080 pixels sensor. Each camera was positioned in front of one of two adjacent aquarium walls (i.e. orthogonally to each other). A C-mount 8 mm focal length lens (VHF8MK) (SPACE Inc., Tokyo, Japan) was mounted on the cameras to realize a field of view to overlook the entire aquarium at ∼250 mm distance. A longpass filter (LP550) (Midwest Optical Systems Inc., Palatine, USA) with an absorption range of 200–545 nm, transmission region of 550–1100 nm and 550 nm cut-off wavelength was mounted on both lenses (Figure 1a–c). This allowed for the fluorescent signal from the quantum dots (585 nm and 655 nm) to pass non-attenuated through the filter while the blue excitation light (435–490 nm) was completely blocked. The cameras were set to record videos at 10 frames per second.

### Excitation Lights

Four custom made excitation light sources were mounted on top of the experimental aquarium using blue light emitting diode (LED) arrays with a peak and dominant wavelength of 465 nm and 470 nm respectively (ENFIS UNO Tag Array Blue 465 nm) (ENFIS LIMITED, Swansea, United Kingdom). Each excitation light source was equipped with a bandpass filter (BP470) (Midwest Optical Systems Inc., Palatine, USA) with an absorption range of 270–338 nm, reflection region of 510–1100 nm and pass band wavelength at 435–490 nm.

As organisms show strong behavioural response to ultraviolet radiation (UV), an UV source was incorporated to the same construction as the excitation lights with the exception that we here used a UVA LED array (ENFIS UNO Tag Array Ultra-Violet (UVA) 375 nm) (ENFIS LIMITED, Swansea, United Kingdom) with a peak wavelength of 375 nm. This set-up allows for mimicking of an UV threat.

### Software

In order to control cameras, lamps and to record synchronized videos, we developed a custom Microsoft Windows application called STEVICORD (stereo video recording) using National Instruments LabView 2011 in combination with National Instruments Vision Development Module. The application was used to synchronize and record the images from the two cameras as uncompressed videos in order to keep as much of the information in the video as possible. Although we have here used our custom made software, any stereo recording software can be used for this set-up (see e.g. ref. [6], [16]), making the method generally applicable.

The LED light sources were connected to a four-channel programmable power supply (Hameg HMP4040) (Hameg, Mainhausen, Germany). Two channels were used to power four excitation lights with a fixed voltage and current of 16 V and 1.5 A (24 W) for each excitation lamp, providing enough light for excitation in the whole water column. The two remaining channels were used for controlling one UV source and the other was left available for future experimental applications.

### Image Processing and Tracking

The videos were compressed up to 75% using a macro script in ImageJ 1.45 software [17] as Portable Network Graphics (PNG) lossless images and saved in a new video file.

To obtain the zooplankters 3D swimming trajectories, the experimental videos were processed using a second ImageJ macro script. This macro processes the videos following four steps: (1) segmenting each frame into objects of interest (i.e. the organism) and background, (2) determining – for both cameras – the 2D tracks of all recorded organisms, (3) getting the colour information of the tracked organisms, (4) assigning each 2D track from the first camera with the corresponding 2D track from the second camera into 3D trajectories [18].

**Figure 2:**
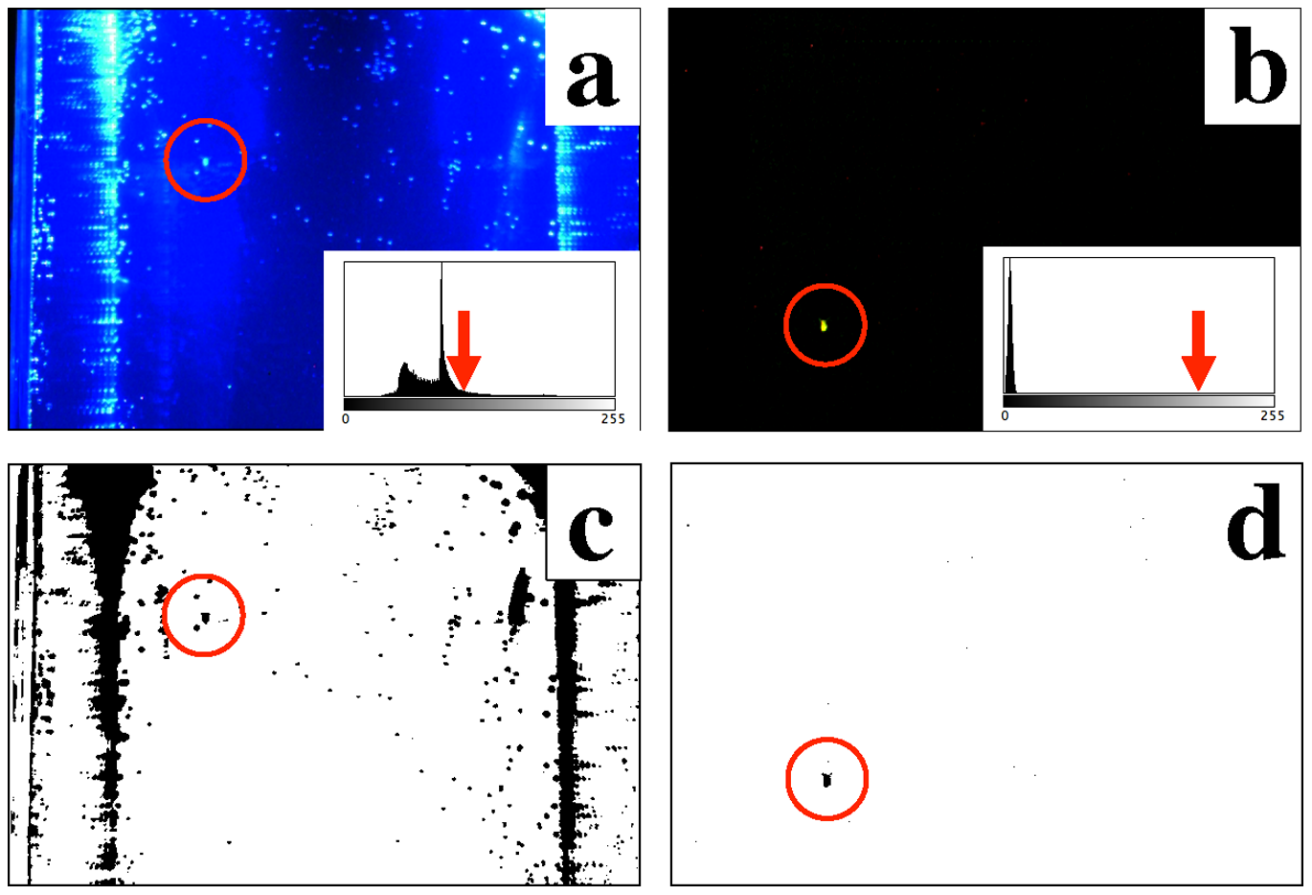
Comparison of image quality using traditional (unlabelled) and Q-dot labelled organisms (*D. magna*). Video frame crop of 600×400 pixel representing a cross-section of the aquarium for (a) unlabelled and (b) quantum dot labelled (585 nm) *Daphnia*, and respective binary images obtained by a fixed threshold value (c and d, respectively). Inlets are the frame luminosity histogram and red arrows indicate the *Daphnia* mean luminosity value. *Daphnia* position is indicated by red circles.

In the first step, each frame was converted in a binary image using upper and lower threshold values manually chosen for each experiment. To remove isolated pixels, i.e. noise, an open morphological filter was applied on each binary frame (see ref. [19]). The number of iterations and number of adjacent background pixels used by the filter was manually set for each experiment based on the residual noise present in the image. This in combination with the quantum dot labelling ensures a good detectability and isolation of the organism as well as reduces the noise in the images (see Figure 2 for example of the binary converted image with applied threshold on a labelled and unlabelled *D. magna*).

In the second step the 2D tracks were obtained using a modified version of ImageJ MTrack2 particle tracking plugin [20], which identified all the particles in each frame and their identity in each successive frame using the nearest neighbour distance. The particles minimum and maximum dimensions, their maximum distance travelled per frame, and the minimum track length were passed by our macro to the plugin.

The third step determines which quantum dot (i.e., red or yellow) that was used for labelling the tracked organism. The fundamental colours red, green and blue (RGB) of each particle was obtained at the centroid position coordinate determined by the MTrack2 plugin. A G/R ratio of 0 implied a red particle and a G/R ratio of ∼1 implied a yellow particle. This information was later used when evaluating and assembling the tracks.

Finally the 2D tracks obtained from both cameras were assigned to each individual. The decision of whether two 2D tracks were from the same organism was based on the root mean square (RMS) difference between the common z-axis coordinates of all possible combinations. The two 2D tracks, for which the RMS value was minimal and below a certain user-defined threshold, were assembled.

As unlabelled organisms could not be detected by the automated tracking macro we had to use a 2D manual tracking macro where the user, for each frame, registered the coordinates of the organism by marking its position. The coordinates were then manually put together into 3D trajectories.

### Proof of Principle

As a first proof-of-principle demonstration of our tracking method we monitored and tracked the response of two *D. magna* in an aquarium with dimensions 0.15×0.15×0.6 m to ultra violet (UV) exposure *– D. magna* is known to respond with a negative phototaxis when exposed to UV radiation [21]. To that end we coated the two individuals with poly-L-lysine-coated quantum dots that fluoresce at 585 nm (yellow) and 655 nm (red), respectively. Using our synchronized multiple-camera setup the individual organisms were simultaneously tracked both in the presence and absence of UV radiation.

### Validation Experiment – UV Response Experiment

To evaluate the labelling protocol and the tracking method we used *D. magna* fed with the green algae *Scenedesmus sp.* two times per week. *Daphnia magna* are known to have a phototactic behaviour showing both positive and negative phototaxis depending on which wavelength of light they are exposed to [21]. When exposed to wavelengths at the UV-A region (315–400 nm), *Daphnia* show a strong negative phototaxis [21].

Initially we labelled 18 *D. magna* with poly-L-lysine conjugated quantum dots fluorescent at 585 nm (yellow, n = 9) and 655 nm (red, n = 9) prepared as described above. A control group of *Daphnia* (n = 9) was treated according to the same protocol as the labelled *Daphnia,* with the exception that 40 µL water was added instead of quantum dot solution.

One *Daphnia* was then transferred to the experimental aquarium containing 11 litres of water (L = 0.15 m, W = 0.15 m, H = 0.6 m) and 250 ml of *Scenedesmus sp.* algal suspension and the excitation light was switched on. The *Daphnia* was allowed to acclimatize in the aquarium for twenty minutes prior to the experiment. Then the recording started and recorded the behaviour of the *Daphnia* during one minute, whereafter the UV source was switched on with a fixed current of 0.1 A, corresponding to approximately 250 µW/cm^2^, and the behaviour was recorded for one additional minute. After the experiment the *Daphnia* was removed from the aquarium and replaced with a new individual. This was repeated seven times for each treatment (i.e. red, yellow and unlabelled).

### Data Evaluation

The trajectories were analysed using R 2.15.2 software [22]. Analysis of multivariate variance (MANOVA) was performed on the individuals mean value of vertical position, speed and distance moved post and pre exposure to UV using the basic R package. The displacement of *Daphnia* was calculated by computing the cumulative sum of the 3D Euclidian distance of each position. The speed was calculated using the central difference method using the distance travelled between the previous and the successive 3D position, divided by the time lag between two consecutive frames (i.e. 0.2 sec).

### Generality

To demonstrate that the labelling method can be generally applicable over a wide range of taxa we also labelled ostracods as well as two types of aquatic insect larvae, one *Chaoborus* larva and one mayfly larva (*Cloeon sp.*). We also labelled 8 *D. magna*, four red and four yellow. These were then placed in the aquaria all at once to test how the tracking macro would handle multiple individuals. All organisms were labelled using the same procedure as described above.

### Ethics Statement

All studies were carried out in accordance to Swedish law and no ethical permits were needed.

## Results

**Figure 3:**
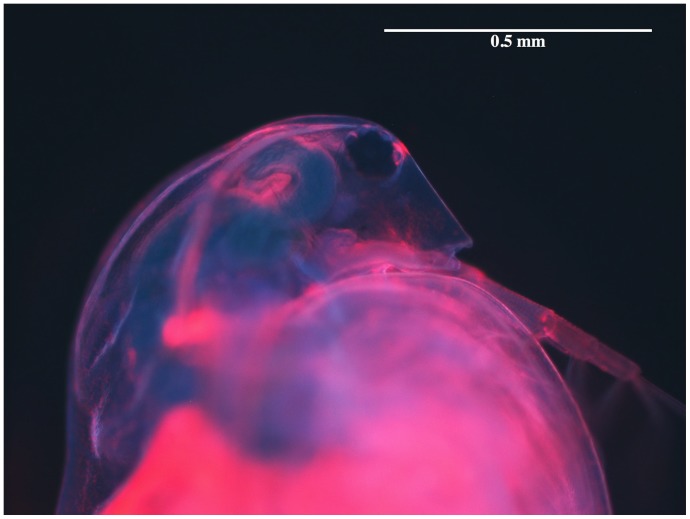
Fluorescence from a quantum-dot-labelled organism. Optical microscope image of a *D. magna* labelled with poly-L-lysine coated quantum dots that fluoresce at 655 nm (red). The blue light in the picture is scattered excitation light. (See also Video S1 for a video of labelled Daphnia magna swimming around.).

The binding of the poly-L-lysine coated quantum dots to the carapace was verified by placing a *D. magna* in a fluorescence microscope (Figure 3) were it is seen that the quantum dots are associated with the outside of the *D. magna* carapace. Some particles can also be seen to have ended up in the gut channel as a result of the continuous filtering of water during the incubation.

### Proof of Principle Study

**Figure 4:**
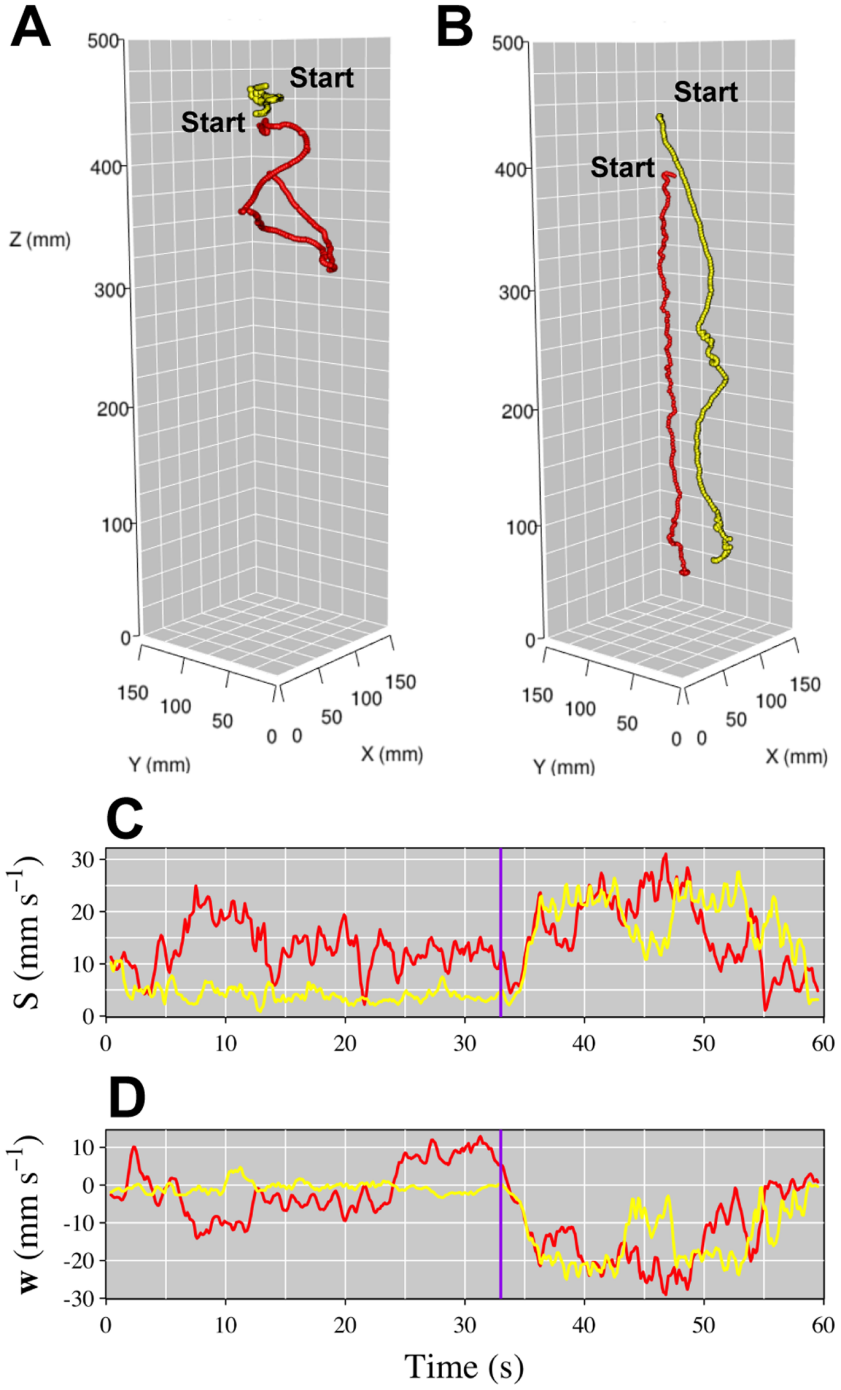
Tracking of the position, speed and vertical displacement of two *D. magna* in the absence and presence of UV radiation. Two individuals were labelled with red and yellow quantum dots, respectively, and monitored simultaneously to obtain 3D trajectories of the two organisms in the absence (a) and presence (b) of UV radiation. From the data we also extracted the 3D swimming speed S (c), and vertical velocity **w** (d). Negative values of **w** indicate a downward displacement, the purple line marks the time at which the UV light was turned on. See also Video S2 for an animation of the UV response (NOTE: The animation is 40 s, i.e. 20 s UV off +20 s UV on).

The proof of principle study showed that when no UV-radiation is present, the *Daphniids* swim randomly in the aquarium (Figure 4a), whereas when the UV-radiation source is switched on, the organisms rapidly respond with negative phototaxis in order to escape the threat from damaging UV radiation (Figure. 4b). Our method also allows us to extract the individuals’ 3D swimming speed (Figure 4c) and vertical velocity (Figure 4d) from the tracking data, from which it is seen that both 3D swimming speed and vertical speed increases as the UV radiation is turned on as a result of the phototactical response.

### Validation Experiment – UV Response Experiment

**Figure 5:**
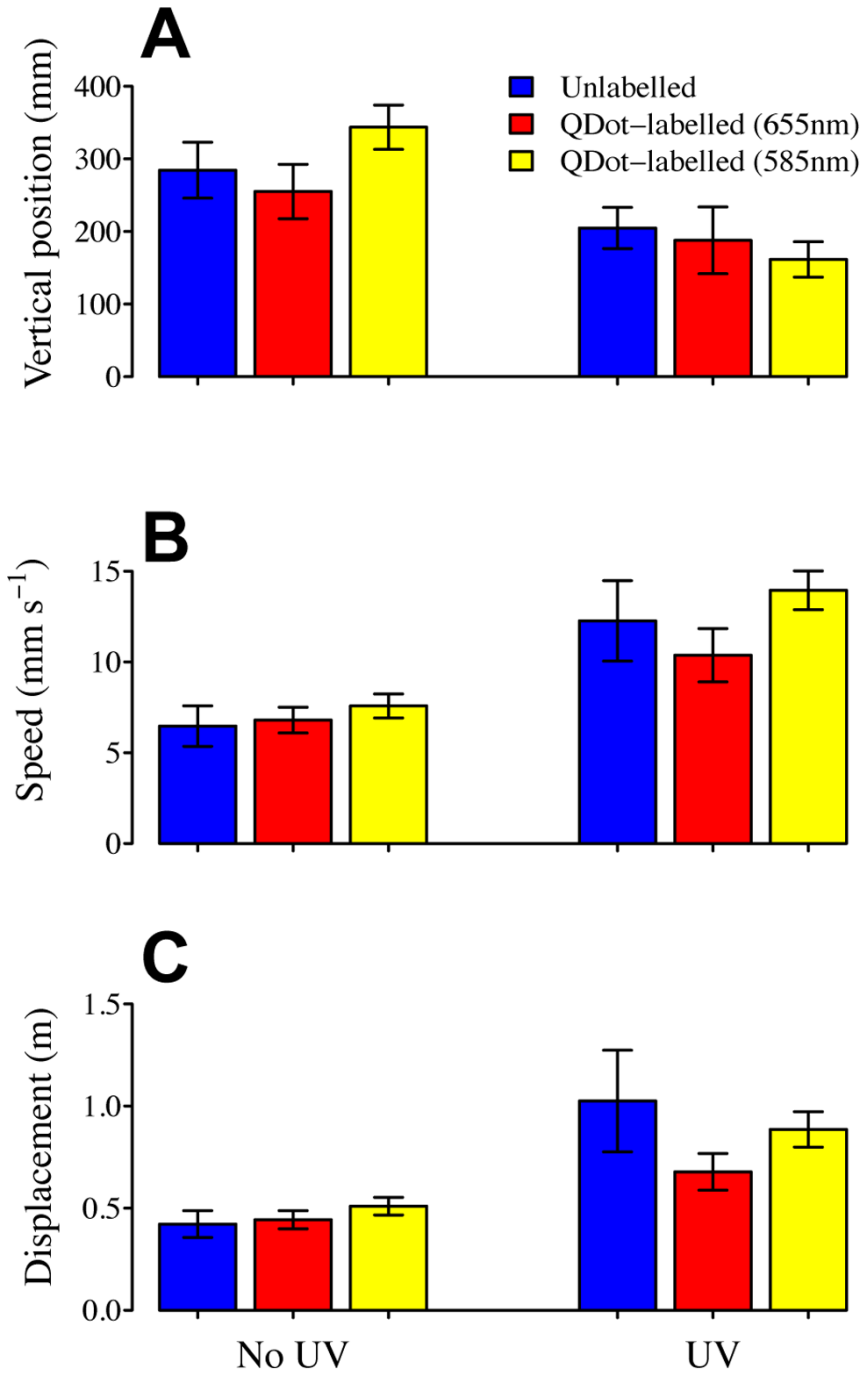
Comparison of the behavioural response of labelled (red and yellow bars) and unlabelled (blue bars) *D. magna* to UV radiation. Swimming performance data for *D. magna* showing mean vertical position (a), speed (b) and displacement (c) pre exposure to UV (left) and during UV exposure (right). The labelled organisms were labelled with quantum dots fluorescent at 655 nm (red bars) and 585 nm (yellow bars). n for all treatments = 7. Error bars show +/−1s.e.m.

There was no significant effect of labelling type on the behaviour of the organisms, i.e. the animals were not affected by the labelling (Figure 5, MANOVA: Wilks’ λ = 0.746, F_6, 68_ = 1.79, p = 0.11). However, there was a significant main effect of UV on the behaviour of the organisms (MANOVA Wilks’ λ = 0.462, F_3, 34_ = 13.17, p<0.001), which were significant for all three variables: vertical position (F_1, 36_ = 14.76, p<0.001), speed (F_1, 36_ = 23.84, p<0.001) and displacement (F_1, 36_ = 18.48, p<0.001). There was no significant interaction between UV exposure and labelling for any of the studied parameters (MANOVA Wilks’ λ = 0.716, F_6, 68_ = 2.05, p = 0.07). All indicating that the labelling constitutes a non-perturbing tracking device for small organisms. The organisms were also found to shed the attached quantum dots as they moulted and then continued to reproduce, indicating that they were unaffected by the experimental procedure (personal observation).

### Generality

In order to test the general applicability of our labelling and tracking method we also applied it on ostracods (see Figure S1 and Video S3) as well as two types of aquatic insect larva, a mayfly larvae (*Cloeon sp.,* see Figure S2 and Video S4) and a *Chaoborus* larva (see Video S5). The same labelling procedure that was used for the *D. magna* was used to label these. Hence, several taxonomically widely separated organisms can be successfully labelled and tracked, suggesting that the method is generally applicable. The multiple tracking approach showed that the tracking macro successfully separated and tracked the eight individuals as well as identified their colour labelling (see Data S1 for tracking database and Video S1 for the video used for the tracking).

## Discussion

The quantum-dot-based nanoparticle labelling and tracking method described here presents a general and novel approach to perform laboratory 3D studies of the movement and behaviour of small organisms in large volumes of observation. While studies on individual zooplankter behaviour have been performed previously (see e.g. ref. [6], [23], [24]), to our knowledge, tracking has so far been limited to multiple tracking of zooplankton in 3D volumes of observation of around 1 litre [7], [8]. Using the quantum dot labelling procedure allows us to utilize a volume of observation of more than 11 litres, as the organisms now can be more easily seen due to the fluorescence emitted by the excited quantum dots (see e.g. Figure 2), thereby opening up for behavioural studies of individual animals in the laboratory at a far more natural scale, for these mm-scale organisms, than previously possible.

Within biology this technique has the potential to advance research fields that have been hampered by the lack of methodology for individual-based tracking of small organisms [25], [26]. One of these fields is zooplankton ecology in which e.g. diel vertical migration constitutes one of the largest daily biomass migrations on Earth [27]. One of the most common approaches when studying vertical migration of zooplankton involves sampling and filtration of several litres of water along a vertical gradient and thereafter counting the organisms [28]. While such studies reasonably well reflect community- or population level behaviour, individual differences in behavioural responses cannot be resolved using such approaches, therefore making it impossible to study parameters such as individual swimming rate and migration or the variation of those parameters among individuals in a population. This is of crucial importance for studying e.g. predator-prey interactions, feeding and mating behaviour, as well as threat avoidance behaviour.

Another crucial advantage of our approach is that it allows for the implementation of highly automated tracking procedures, whereby the individual motion of many mm-scale organisms can be tracked simultaneously with minimal labour. This is in contrast to the manual, optical identification of individual organisms that needs to be employed otherwise (and which was used in the collection of data for the unlabelled *D. magna*’s response to UV-radiation in the comparison with the behavioural response of labelled organisms – Figure 5) and which is very laborious because of the transparent nature of zooplankton [29]. The method did not affect the organism’s behaviour and the observed continued reproduction after exposure to quantum dots is also supported by a previous study showing no effects on the reproduction of *D. magna* after exposure to quantum dots [14]. Combined with the non-perturbative, non-invasive feature of our approach and its general applicability (as shown by our test of generality on several classes of organisms) our quantum-dot-labelling method allows for studies of individual behaviour and interactions among animals of similar or different taxa at the community level.

The novel labelling protocol and tracking system presented here will open up new research fields addressing the behaviour of small organisms that were previously intractable due to methodological constraints. In a broader perspective it is also our belief that this new technique will advance plankton ecology and be applicable for tracking other small organisms, not only in water but also small terrestrial organisms, such as insects. Hence, by combining biology and nanotechnology, tracking of small animal movements and migration can now be raised to the same scientific level as for larger animals, such as birds and fish.

## Supporting Information

Figure S1 **3D tracking of three ostracods.** The three paths correspond to the swimming trajectories covered by three ostracods (labelled with red-fluorescing quantum dots) during a time frame of 10 s (see Video S3 for the corresponding video recordings from the two synchronized cameras).
(TIF)

Figure S2 **Tracking the position and speed of a mayfly larva (** ***Cloeon sp.*** **).** Swimming trajectory (a) and speed (b) of a mayfly larva (labelled with red-fluorescing quantum dots) tracked during a time frame of 10 s in an aquarium with dimensions 0.15×0.15×0.6 m (see Video S4 for the video recordings corresponding to the data used to extract the position and speed).
(TIF)

Video S1 **Simultaneous tracking of several** ***Daphnia magna.*** In this video multiple (n = 8) *D. magna* labelled with red (n = 4) and yellow (n = 4) fluorescing poly-L-lysine coated quantum dots are simultaneously tracked in an aquarium with dimensions 0.15×0.15×0.6 m. Left panel shows the raw video from one of the two cameras and the right panel shows the segmented video from the same camera. Note the easy detection when the video is segmented applying a fixed luminosity threshold. Video length is 10 seconds. See Data S1 for a multi 3D tracking database of this video.
(MOV)

Video S2 **3D-tracking of two** ***Daphnia magna*** **in the absence/presence of UV radiation.** Animation depicts the behavioural response of two *D. magna* (labelled with red and yellow fluorescing poly-L-lysine coated quantum dots, respectively) to UV radiation (the data used in this track is the same as that shown in Fig. 3 of the main text). The UV radiation is introduced after 20 seconds into the tracking.
(MOV)

Video S3 **Recording of quantum-dot-labelled ostracods.** The video shows the recording of three ostracods labelled with red poly-L-lysine coated quantum dots in an aquarium with dimensions 0.15×0.15×0.6 m during 10 seconds. Video has been cropped to focus on the area occupied by the ostracod to ease the detection in this demonstration. The left and right panels show the raw video from camera one and two, respectively.
(MOV)

Video S4 **Recording of a quantum-dot-labelled mayfly larva (** ***Cloeon sp.*** **).** This video shows the recording of a mayfly larva labelled with red poly-L-lysine coated quantum dots during 10 s in an aquarium with dimensions 0.15×0.15×0.6 m. Left panel shows the raw video from one of the two cameras. Right panel shows the segmented video obtained applying a fixed luminosity threshold to the raw video. Video has been cropped to focus on the area occupied by the mayfly larva to ease the detection in this demonstration.
(MOV)

Video S5 **Tracking of quantum-dot-labelled** ***Chaoborus*** **larva.** The video shows the recording of a *Chaoborus* larva labelled with red poly-L-lysine coated quantum dots during 60 s in an experimental aquarium with dimensions 0.15×0.15×0.6 m. Left and right panel shows the raw video from camera one and two, respectively. Video has been cropped to focus on the area occupied by the *Chaoborus* larva to ease the detection in this demonstration.
(MOV)

Data S1 ***Daphnia magna*** **multi 3D tracking.** Example of database output from the simultaneous tracking of multiple *D. magna*. Data is shown for eight individuals, four labelled with red-fluorescing and four labelled with yellow-fluorescing quantum dots. Tracking and computation was performed using ten observations per seconds, data is shown for one position per second. Id denotes individual number, Qdot denotes the colour of the used labelling quantum dot, time refers to time (s) from start of recording, X, Y and Z denotes the coordinates in mm in the aquarium along the X, Y and Z axis, **u** denotes the velocity along the X-axis (mm/s), **v** denotes the velocity along the Y-axis (mm/s), **w** denotes the velocity along the Z-axis (mm/s), speed denotes the three dimensional speed (mm/s), Gross displacement denotes the total distance moved (mm) and Net displacement denotes the distance moved (mm) from the point of origin (starting point). The tracked video can be seen in Video S1.
(PDF)
