# Supplementary figures and images for: Three-Dimensional Tracking of Small Aquatic Organisms Using Fluorescent Nanoparticles
Source: PLoS One. 2013 Nov 7;8(11):e78498. doi: 10.1371/journal.pone.0078498 (PMC3820599; doi:10.1371/journal.pone.0078498)

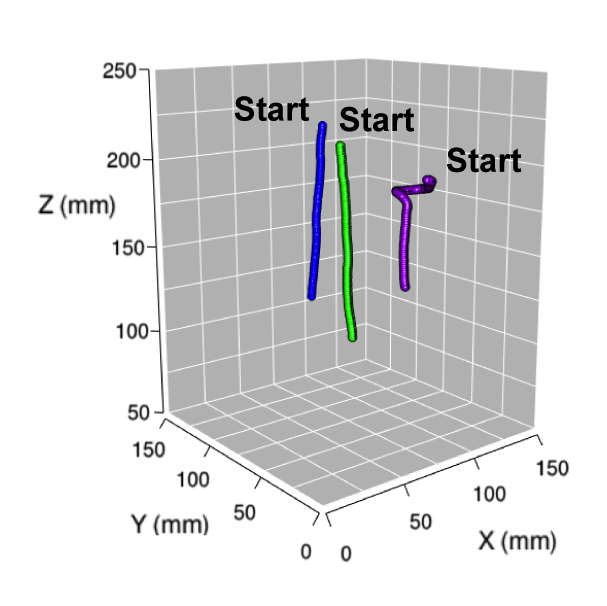

Supplement: Figure S1 — 3D tracking of three ostracods. The three paths correspond to the swimming trajectories covered by three ostracods (labelled with red-fluorescing quantum dots) during a time frame of 10 s (see Video S3 for the corresponding video recordings from the two synchronized cameras). (TIF) [file pone.0078498.s001.tif]

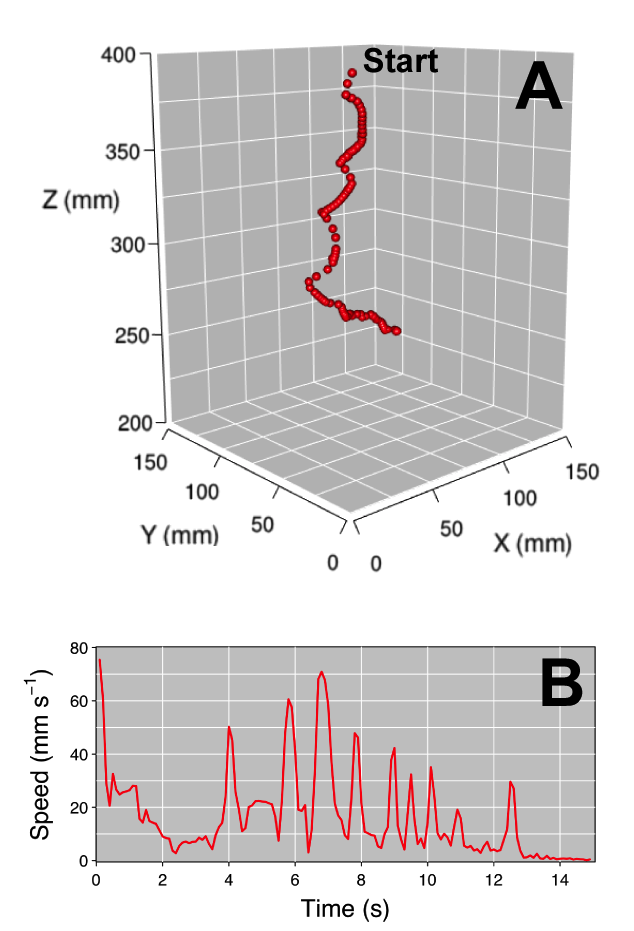

Supplement: Figure S2 — Tracking the position and speed of a mayfly larva ( Cloeon sp. ). Swimming trajectory (a) and speed (b) of a mayfly larva (labelled with red-fluorescing quantum dots) tracked during a time frame of 10 s in an aquarium with dimensions 0.15×0.15×0.6 m (see Video S4 for the video recordings corresponding to the data used to extract the position and speed). (TIF) [file pone.0078498.s002.tif]
