# Supplementary material for: Three-Dimensional Tracking of Small Aquatic Organisms Using Fluorescent Nanoparticles
Source: PLoS One. 2013 Nov 7;8(11):e78498. doi: 10.1371/journal.pone.0078498 (PMC3820599; doi:10.1371/journal.pone.0078498)
Supplement: Data S1 — Daphnia magna multi 3D tracking. Example of database output from the simultaneous tracking of multiple D. magna. Data is shown for eight individuals, four labelled with red-fluorescing and four labelled with yellow-fluorescing quantum dots. Tracking and computation was performed using ten observations per seconds, data is shown for one position per second. Id denotes individual number, Qdot denotes the colour of the used labelling quantum dot, time refers to time (s) from start of recording, X, Y and Z denotes the coordinates in mm in the aquarium along the X, Y and Z axis, u denotes the velocity along the X-axis (mm/s), v denotes the velocity along the Y-axis (mm/s), w denotes the velocity along the Z-axis (mm/s), speed denotes the three dimensional speed (mm/s), Gross displacement denotes the total distance moved (mm) and Net displacement denotes the distance moved (mm) from the point of origin (starting point). The tracked video can be seen in Video S1. (PDF) [file pone.0078498.s008.pdf]

id	QDot	time (s)	x (mm)	y (mm)	z (mm)	u (mm/s)	v (mm/s)	w (mm/s)	speed (mm/s)	Gross displacement (mm)	Net displacement (mm)
1	red	0	66.13	84.21	388.88	NA	NA	NA	NA	0	0
1	red	1	73.08	86.65	396.16	0.66	0.04	0.62	0.91	10.6	10.36
1	red	2	82.33	84.04	404.51	0.72	-0.55	0.68	1.13	23.51	22.51
1	red	3	90.54	84.67	414.72	1.3	0.73	1.56	2.16	37.67	35.55
1	red	4	101.3	90.11	427.48	0.95	0.38	1.15	1.54	55.33	52.55
1	red	5	112.79	86.01	439.77	1.24	-0.77	0.94	1.73	73.73	69.07
1	red	6	120.33	79.63	442.92	0.27	-0.04	-0.09	0.29	87.49	76.67
1	red	7	120.88	76.39	446.79	-0.34	0	0	0.34	96.92	80.08
1	red	8	119.75	76.89	445.45	0	0.88	0	0.88	102.35	78.29
1	red	9	118.8	89.81	439.64	-0.52	2.1	0.88	2.33	118.53	73.36
2	red	0	111.49	91.24	240.3	NA	NA	NA	NA	0	0
2	red	1	99.44	89.64	225.75	-1.17	0.84	-1.37	1.99	25.18	18.96
2	red	2	86.93	90.07	212.05	-1.46	-0.91	-1.98	2.62	44.33	37.45
2	red	3	74.91	80.89	198.71	-1.16	-1.66	-0.71	2.15	65.36	56.35
2	red	4	69.1	69.63	187.14	0.26	-1.55	-2.23	2.73	84.55	71.34
2	red	5	70.22	64.2	174.03	1.12	-1.05	-1.99	2.51	100	82.62
2	red	6	79.67	57.84	153.97	1.11	-1.16	-2.69	3.13	123.27	97.88
2	red	7	83.27	50.38	137.52	-1.37	-0.9	-1.08	1.96	143.76	114.15
2	red	8	84.34	46.49	119.27	0.06	0.17	-2.07	2.08	164.9	131.86
2	red	9	91.57	51.8	98.51	-0.04	1.23	-1.75	2.14	188.98	148.52
3	red	0	44.19	106.43	216.47	NA	NA	NA	NA	0	0
3	red	1	50.52	97.28	215.9	0.87	-0.95	-0.26	1.31	11.87	11.14
3	red	2	61.31	94.02	217.89	0.86	-0.1	0.09	0.87	23.92	21.19
3	red	3	66.52	85.36	221.59	-0.37	-1.96	0.65	2.1	37.3	31.13
3	red	4	69.42	69.95	224.7	0.49	-1.84	0.33	1.93	53.67	45.11
3	red	5	66.51	57.77	227.3	-0.51	-0.93	0.31	1.11	67.28	54.62
3	red	6	60.34	57.71	231.25	-0.37	0.29	0.33	0.57	75.86	53.41
3	red	7	66.36	60.43	238.5	0.27	-0.81	0.49	0.98	88.82	55.61
3	red	8	69.9	49.24	243.73	1	-1.39	0.95	1.96	102	68.37
3	red	9	79.14	37.7	248.81	0.2	-1.28	0.91	1.59	117.84	83.61
4	red	0	32.1	112.57	124.53	NA	NA	NA	NA	0	0
4	red	1	31.8	111.81	125.33	0.24	0.35	-0.07	0.43	5.47	1.14
4	red	2	30.91	113.48	125.67	0.08	-0.77	-0.25	0.81	11.93	1.88
4	red	3	33.04	113.98	125.12	0.62	-0.07	-0.7	0.93	17.14	1.79
4	red	4	31.23	114.13	124.79	-0.07	-0.39	-0.03	0.4	23.54	1.81
4	red	5	31.38	113.34	126.56	-0.34	0.29	0.85	0.96	26.72	2.29
4	red	6	32.03	112.85	125.09	0.48	-0.23	0.16	0.56	32.07	0.63
4	red	7	33.44	113.35	124.25	0.05	-0.07	-0.07	0.11	37.68	1.58
4	red	8	32.28	114.6	124.4	0.16	0.01	-0.3	0.33	41.92	2.04
4	red	9	31.72	114.74	125.18	0.42	0.69	-0.06	0.81	50.07	2.3

5	yellow	0	135.03	10.58	492.79	NA	NA	NA	NA	0	0
5	yellow	1	133.26	9.38	491.75	-0.15	-0.24	-0.09	0.3	5.53	2.38
5	yellow	2	133.2	12.12	493.61	-1.08	0.13	-0.47	1.18	13.36	2.53
5	yellow	3	134.6	9.99	494.34	-0.32	0.72	-0.1	0.8	20.88	1.71
5	yellow	4	133.29	13.48	491.76	0.33	-0.15	0	0.36	27.71	3.54
5	yellow	5	134.2	9.88	487.41	-0.04	0.21	-0.33	0.4	36.33	5.49
5	yellow	6	134.2	13.3	484.12	0.03	0.14	-0.5	0.52	42.24	9.12
5	yellow	7	134.31	14.29	480.65	0.03	0.22	0	0.22	46.71	12.71
5	yellow	8	134.02	15.3	478.12	-0.2	0.04	0.28	0.35	50.87	15.44
5	yellow	9	134.54	15.18	476.44	0.17	0.02	-0.49	0.51	55.27	16.99
6	yellow	0	129.53	37.49	187.8	NA	NA	NA	NA	0	0
6	yellow	1	129.42	35.58	176.15	-0.93	0.2	-0.66	1.16	17.58	11.81
6	yellow	2	134.2	29.04	164.75	0.18	-0.44	-1.44	1.51	37.67	24.99
6	yellow	3	128.17	34.7	154.41	-1.66	-0.16	-1.17	2.03	54.12	33.53
6	yellow	4	131.09	22.95	143.4	-0.5	-0.74	-0.76	1.18	72.82	46.75
6	yellow	5	134.56	21.04	129.88	-0.41	-0.12	-0.52	0.68	89.31	60.42
6	yellow	6	128.58	20.67	130.43	1.02	-0.01	0	1.03	98.88	59.79
6	yellow	7	124.4	23.84	116.93	-0.95	0.24	-1.37	1.68	116.72	72.35
6	yellow	8	121.5	23.55	105.52	-0.59	1.17	-0.83	1.55	130.18	83.84
6	yellow	9	123.7	23.4	90.32	0.98	0.78	-0.48	1.34	149.93	98.67
7	yellow	0	84.05	98.59	98.72	NA	NA	NA	NA	0	0
7	yellow	1	87.04	98.88	96.7	0.19	0.02	-0.17	0.26	8.13	3.62
7	yellow	2	86.26	102.3	97.25	-0.33	-0.05	-0.12	0.35	16.22	4.56
7	yellow	3	85.06	103.45	97.54	0.11	0.57	0.17	0.6	18.94	5.1
7	yellow	4	89.59	107.59	95.28	0.03	0.05	0.09	0.1	26.45	11.11
7	yellow	5	87.1	108.45	96.46	-0.03	0.01	-0.05	0.06	30.05	10.57
7	yellow	6	84.89	108.03	98.01	-0.4	0.06	0.24	0.47	38.43	9.5
7	yellow	7	83.81	108.69	99.05	-0.44	0.58	0.15	0.74	42.36	10.11
7	yellow	8	80.54	109.7	100.32	0.02	-0.02	-0.12	0.12	49.72	11.76
7	yellow	9	79.54	110.66	101.35	-0.4	0.97	-0.27	1.08	55.99	13.15
8	yellow	0	122.19	19.63	80.73	NA	NA	NA	NA	0	0
8	yellow	1	121.08	19.57	81.9	-0.77	-0.18	1.16	1.4	3.44	1.61
8	yellow	2	120.23	14.06	81.95	0.45	-0.53	-0.5	0.86	14.93	6.03
8	yellow	3	123.55	11.3	78.38	0.35	0.13	-0.44	0.57	21.4	8.76
8	yellow	4	126.44	11.54	75.3	0.1	0.02	-0.1	0.14	26.43	10.63
8	yellow	5	127.12	11.13	74.94	0.11	-0.05	-0.02	0.12	27.9	11.41
8	yellow	6	126.9	11.14	74.9	0.05	0.04	0	0.07	29.68	11.32
8	yellow	7	126.67	11.84	74.44	0.06	0.05	-0.11	0.13	30.93	10.97
8	yellow	8	126	11.63	75.79	0.02	0.03	-0.1	0.1	32.62	10.14
8	yellow	9	125.73	11.9	75.63	-0.07	0.02	-0.01	0.07	33.68	9.91
